# Cerebrospinal Fluid Parameters in Antisense Oligonucleotide-Treated Adult 5q-Spinal Muscular Atrophy Patients

**DOI:** 10.3390/brainsci11030296

**Published:** 2021-02-26

**Authors:** Lars Hendrik Müschen, Alma Osmanovic, Camilla Binz, Konstantin F. Jendretzky, Gresa Ranxha, Paul Bronzlik, Omar Abu-Fares, Flavia Wiehler, Nora Möhn, Martin W. Hümmert, Stefan Gingele, Friedrich Götz, Martin Stangel, Thomas Skripuletz, Olivia Schreiber-Katz, Susanne Petri

**Affiliations:** 1 Department of Neurology, Hannover Medical School, 30625 Hannover, Germany; osmanovic.alma@mh-hannover.de (A.O.); Binz.Camilla@mh-hannover.de (C.B.); Konstantin.Jendretzky@stud.mh-hannover.de (K.F.J.); Ranxha.gresa@mh-hannover.de (G.R.); Wiehler.Flavia@mh-hannover.de (F.W.); moehn.nora@mh-hannover.de (N.M.); huemmert.martin@mh-hannover.de (M.W.H.); gingele.stefan@mh-hannover.de (S.G.); stangel.martin@mh-hannover.de (M.S.); skripuletz.thomas@mh-hannover.de (T.S.); Schreiber-Katz.Olivia@mh-hannover.de (O.S.-K.); petri.susanne@mh-hannover.de (S.P.); 2 Department of Diagnostic and Interventional Neuroradiology, Hannover Medical School, 30625 Hannover, Germany; Bronzlik.paul@mh-hannover.de (P.B.); Abu-Fares.omar@mh-hannover.de (O.A.-F.); Goetz.friedrich@mh-hannover.de (F.G.)

**Keywords:** spinal muscular atrophy (SMA), nusinersen, antisense oligonucleotide (ASO), cerebrospinal fluid (CSF), lumbar puncture

## Abstract

Approval of nusinersen, an intrathecally administered antisense oligonucleotide, for the treatment of 5q-spinal muscular atrophy (SMA) marked the beginning of a new therapeutic era in neurological diseases. Changes in routine cerebrospinal fluid (CSF) parameters under nusinersen have only recently been described in adult SMA patients. We aimed to explore these findings in a real-world setting and to identify clinical and procedure-associated features that might impact CSF parameters. Routinely collected CSF parameters (leukocyte count, lactate, total protein, CSF/serum albumin quotient (QAlbumin), oligoclonal bands) of 28 adult SMA patients were examined for up to 22 months of nusinersen treatment. Total protein and QAlbumin values significantly increased in the first 10 months, independent of the administration procedure. By month 14, no further increases were detected. Two patients developed transient pleocytosis. In two cases, positive oligoclonal bands were found in the beginning and in four patients throughout the whole observation period. No clinical signs of inflammatory central nervous system disease were apparent. Our data confirm elevated CSF total protein and QAlbumin during nusinersen treatment. These alterations may be caused by both repeated lumbar punctures and the interval between procedures rather than by the medication itself. Generally, there were no severe alterations of CSF routine parameters. These results further underline the safety of nusinersen therapy.

## 1. Introduction

With U.S. Food and Drug Administration (FDA) approval of the antisense oligonucleotide (ASO) nusinersen, the first disease-modifying treatment for 5q-spinal muscular atrophy (SMA) has become available [1]. SMA is an inherited neuromuscular disorder leading to a broad spectrum of clinical phenotypes (SMA types 0/1–4) with slowly progressive muscle weakness and atrophy [2,3]. The majority of patients carry a homozygous deletion of exon 7 and/or 8 of the survival motor neuron (*SMN*)1 gene, resulting in degeneration of anterior horn cells in the spinal cord and motor nuclei of the lower brainstem. The almost identical *SMN2* gene, which is commonly present in several copies, differs only in a single nucleotide. This change causes the disruption of an exonic splice enhancer in exon 7, and therefore, the gene encodes only low levels of the functional SMN protein [4]. ASOs are small single-stranded nucleic acid polymers that can modulate the gene expression via various mechanisms [5]. ASO-based therapies in SMA modify splicing of *SMN2* at the pre-mRNA level and thereby increase the production of the functional, full-length SMN protein [6]. Nusinersen has been shown to effectively target the central nervous system and substantially prolong survival and improve motor function in SMA patients [7,8]. A major disadvantage of ASOs is their inability to penetrate the blood–brain barrier [5]. Hence, nusinersen must be repeatedly administered intrathecally by lumbar puncture (LP) on days 0, 14, 28, and 63 (loading period), followed by injections every four months (maintenance period). Phase 1 and 2 clinical trials have revealed no safety concerns regarding cerebrospinal fluid (CSF) routine parameters such as total cell count, protein, glucose, inflammatory cytokines, and anti-nusinersen antibodies [7,9]. In a cohort of 25 adolescent and adult SMA type 2 and 3 patients, a slight increase in total CSF protein and CSF/serum albumin quotient (QAlbumin) was detected after the loading period on day 63 compared to baseline [10]. In a larger cohort of 63 SMA patients, this observation was confirmed over a longer treatment period [11]. Besides CSF protein alterations, routine CSF analysis revealed the presence of inclusions in macrophages during nusinersen treatment [12]. A potential impact of repeated LP on CSF protein elevation was suggested by Wurster and colleagues [11]. A slowdown of CSF flow throughout the therapy was hypothesized, though no clinical signs of hydrocephalus were found. However, data on changes in CSF routine parameters in ASO-treated patients against the background of repeated LPs are still limited. It has not been elucidated so far whether the above-mentioned findings are reproducible in an independent adult SMA cohort and whether CSF routine parameter alterations are associated with the treatment or with repeated LP. The objective of our study therefore was to gain further knowledge of the potential impact of nusinersen or its administration on CSF routine parameters and the tolerability of nusinersen according to CSF parameters in SMA patients in a real-world setting. We present the following study in accordance with the Strengthening the Reporting of Observational Studies in Epidemiology (STROBE) reporting checklist [13].

## 2. Materials and Methods

### 2.1. Standard Protocol Approvals, Registrations, and Patient Consents

This study was approved by the local ethics committee of the Hannover Medical School (approval number 2413-2014). All participants gave written informed consent to participate in the study and publication of pseudonymous cohort data before study enrollment.

### 2.2. Participants and Sampling

In this prospective, single-center, observational study, CSF and serum samples of patients with confirmed 5q-associated SMA treated with nusinersen at the Department of Neurology at the Hannover Medical School were collected between November 2017 and December 2019. Clinical parameters and demographics such as age, disease duration, history of scoliosis and spondylodesis, SMA type (2–4), and *SMN2* copy number were collected at baseline. The route of nusinersen administration was either by conventional LP or computed tomography (CT)-guided LP. In this study, only CT-guided LP instead of fluoroscopy-guided LP was performed due to better tolerability and feasibility [14]. The motor function outcome was measured by physiotherapists at baseline and each treatment visit using the Revised Upper Limb Module (RULM) score [15] and the Hammersmith Functional Motor Scale Expanded (HFMSE) [16]. The RULM comprises 20 items rating the arm function, totaling 37 points. Higher scores indicate better function. Specifically designed and validated for SMA patients, the HFMSE contains 33 items and captures mainly gross motor function of major joints and body regions. The score for each item ranges from 0 to 2, with a maximum of 66 points. Additionally, the six-minute walk test (6MWT) was recorded in ambulatory patients at each treatment time point [17]. Side effects, including LP-related adverse events (i.e., cephalgia, neck pain, back pain, dizziness, and nausea), were evaluated at each treatment time point by a direct patient interview. CSF and serum samples were collected before nusinersen injections at the following day (d) or month (m) of treatment: d0, d14, d28, d63, m6, m10, m14, m18, and m22. The following CSF and serum parameters were routinely determined: leukocyte count; erythrocytes; total protein; lactate; CSF/serum albumin quotient (QAlbumin); CSF/serum immunoglobulin G (IgG), immunoglobulin A (IgA), and immunoglobulin M (IgM) quotients; and oligoclonal bands (OCB).

### 2.3. Sample Analysis

All CSF and serum samples were collected before nusinersen administration and were further analyzed within one hour after LP. Determination of the CSF leukocyte count and protein analysis of CSF and corresponding serum samples were performed, as previously described [18]. Briefly, the CSF leukocyte count was manually determined with a Fuchs–Rosenthal counting chamber. A CSF leukocyte count of >5 cells/µL was considered pathologically increased. CSF cytology specimens of nusinersen-treated SMA patients have previously been published and were not part of this study [12]. A cell-free supernatant was used for protein analysis, and the protein concentration of the CSF was assessed by the Bradford dye-binding procedure (Beckmann Coulter DU730 Spectrophotometer, Brea, CA, USA). Albumin and immunoglobulins in the CSF and serum were determined by kinetic nephelometry (Beckman Coulter IMMAGE). For evaluation of the blood–CSF barrier function, the age-adjusted upper limit of QAlbumin was used according to the formula QAlbumin = 4+ (age in years/15 × 10^−3^). Intrathecal immunoglobulin synthesis for IgG, IgA, and IgM was measured regarding Reiber’s revised hyperbolic function referring IgG, IgA, and IgM quotients to QAlbumin [19]. OCB were assessed by isoelectric focusing in polyacrylamide gels with consecutive silver staining [20]. OCB types 2 and 3—both indicating IgG synthesis—were summarized as positive findings. Since contamination by erythrocytes causes false results of CSF routine parameter analysis regarding the cell count, total protein, or QAlbumin, all samples with an erythrocyte count of >2500/µL were excluded [18].

### 2.4. Statistical Analysis

All statistical analyses were performed using either IBM SPSS Statistics version 26 (SPSS Inc., Chicago, IL, USA) or GraphPad Prism 7.0 (GraphPad Software Inc., San Diego, CA, USA). According to the Shapiro–Wilk test, values for the CSF cell count, CSF total protein, QAlbumin, and OCB were not normally distributed. Therefore, the Wilcoxon signed-rank test was used for comparison of CSF values within the SMA patient group and at every treatment time point. Additionally, we stepwise excluded SMA patients with outlying CSF protein parameters (*n* = 1, S16) and SMA patients with CSF–blood barrier dysfunction at baseline (*n* = 5, S00, S04, S06, S10, S19) and compared CSF total protein and QAlbumin values by using the Wilcoxon signed-rank test. We used the Mann–Whitney U-test to examine the influence of CT-guided LP at every time point. We analyzed associations between CSF routine parameters and clinical parameters (age, RULM, HFMSE, 6MWT) by using Spearman’s correlation coefficient (rho). For investigating the associations between SMA type, LP method, *SMN2* copy number, side effects, and CSF routine parameters, the point-biserial correlation coefficient (rPB) was used. To investigate the association between sex, LP technique, OCB status, and procedure-related side effects, the chi-square test was used. The phi-coefficient (phi) was used to describe the effect size. We applied a linear mixed model with restricted maximum likelihood analysis, with treatment time points d0, d14, d63, m6, m10, m14, and m18 as repeated measurements. With either protein concentration or QAlbumin as a dependable variable, we controlled other factors such as age, gender, or LP method, which may be confounding in univariate analysis. Additionally, estimated marginal means were calculated for the treatment time point, gender, and LP method. For post hoc multiple comparisons, *p*-values were corrected by Bonferroni’s procedure. For all statistical tests, a two-tailed significance level of *p* < 0.05 was considered statistically significant. Because of the explorative character of this study, all statistical test results were interpreted as hypothesis generating and not as confirmatory. No adjustment for multiple testing was done. Missing data of CSF routine parameters or incomplete analysis of any participant’s CSF were not taken into account for this calculation.

## 3. Results

### 3.1. Participants’ Characteristics

Demographic data of participants are summarized in Table 1. Twenty-eight adult SMA patients (SMA type 2, *n* = 10; SMA type 3, *n* = 17; SMA type 4, *n* = 1) with a median age of 36 years (range 19–65 years) were enrolled in this study. The one SMA type 4 patient was further analyzed together with SMA type 3 patients. The majority of patients were male (64.3%). Almost every second patient (46.4%) underwent CT-guided LP. The reason for CT-guided LP in the majority of cases was scoliosis (84.6%). In two patients, CT-guided LP was performed after conventional LP had failed beforehand. In seven SMA patients who underwent CT-guided LP for nusinersen treatment, transforaminal injections were necessary at least once during the treatment course. The median scores for the HFMSE and RULM at d0 were 9.5 (range 0–63) and 18.5 (range 0–37), respectively. Ambulatory patients reached 390 meters in the 6MWT in the median (range 0–512 m). It took on average 32.5 years (range 2–62.5 years) from symptom onset to initiation of nusinersen therapy. Two patients discontinued the therapy after 6 and 14 months, respectively. No side effects were reported in 58% of all conducted LP (*n* = 179). Reports on side effects were missing for three LPs. Reported side effects were mainly (80.2%) classified as procedure related.

### 3.2. CSF Routine Parameters

**Figure 1:**
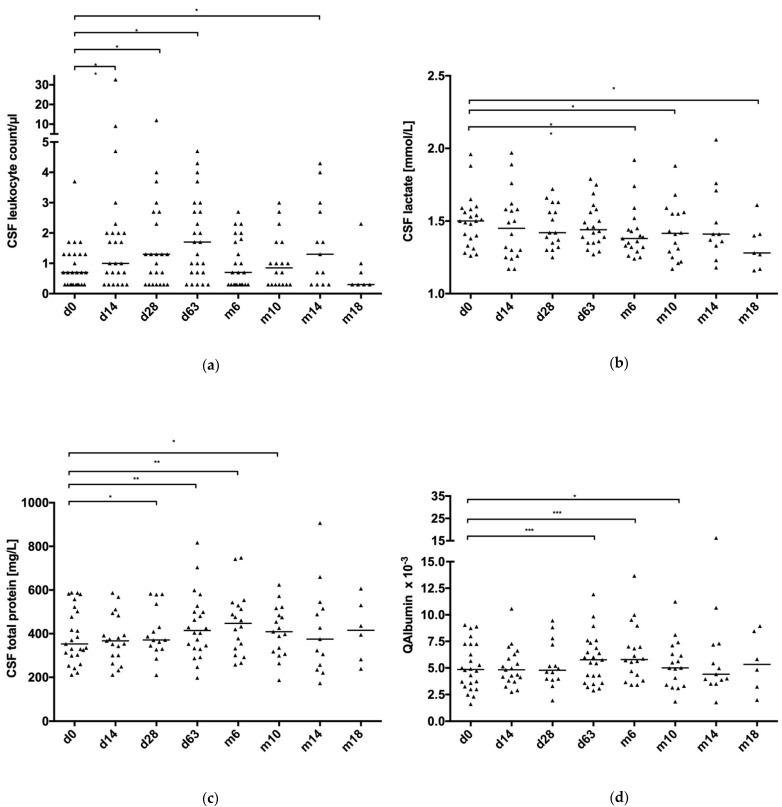
CSF routine parameters during nusinersen therapy (d0 to m18). (**a**) CSF leukocyte count and lactate, (**b**) CSF lactate, (**c**) CSF total protein, and (**d**) QAlbumin. Values displayed as scatter plots with the median. CSF: cerebrospinal fluid; d: day; m: month; QAlbumin: CSF/serum albumin quotient. *: *p* ≤ 0.05; **: *p* ≤ 0.01; ***: *p* ≤ 0.001.

CSF routine parameters of SMA patients were available for baseline (d0; *n* = 28), d14 (*n* = 28), d28 (*n* = 26), d63 (*n* = 27), m6 (*n* = 24), m10 (*n* = 22), m14 (*n* = 15), m18 (*n* = 7), and m22 (*n* = 2). In total, 179 CSF samples of 28 patients were collected. Data were missing for leukocyte count (*n* = 3 samples), lactate (*n* = 33), total protein (*n* = 33 samples), and QAlbumin (*n* = 34 samples) due to various reasons (i.e., too little CSF volume, missing protein diagnostics, etc.). After blood correction by excluding all CSF samples with an erythrocyte count above 2500 cells/µL, 161 CSF leukocyte count values, 130 CSF lactate values, 136 CSF total protein values, and 135 QAlbumin values were analyzed. Since data for m22 were only available for two patients, this time point was not included in the calculation. CSF/serum quotients of IgG, IgA, and IgM were not analyzed in this study, because intrathecal immunoglobulin synthesis was found only in one SMA patient. In Table 2, CSF routine parameters of SMA patients are displayed as the median and range, as well as the total number of analyzed samples. Figure 1 demonstrates CSF routine parameters as box plots.

#### 3.2.1. CSF Leukocyte Count and Oligoclonal Bands

Mild CSF pleocytosis (9/µL) was found in a 36-year-old SMA type 2 female patient (S11) at d14, i.e., two weeks after the first nusinersen administration. The LP was conducted conventionally. No intrathecal immunoglobulin synthesis according to the revised hyperbolic function by Reiber or CSF-specific OCB was found. Subsequently, the leukocyte count was <5/µL during further treatment (d28 to m22). In the CSF of a 32-year-old SMA type 3 male patient (S22), an elevated cell count was found at d14 (33/µL) and d28 (12/µL). The LP was conducted via CT guidance by the standard interlaminar approach. At d0, the CSF leukocyte count was normal (1/µL). At m10, CSF pleocytosis was no longer detectable. However, intrathecal IgA synthesis was found at m10. At every time point (d0 to m10), weakly positive OCB were found in this patient.

In the second of the two patients with positive OCB at baseline, a 51-year-old SMA type 2 female patient (S02), OCB were also positive at both d0 and d14 to m6 as well as m14 to m18. Only at m10, no positive OCB were found. No intrathecal immunoglobulin synthesis (IgG, IgA, IgM) according to Reiber graphs and increased leukocyte count were present at any time point.

In two patients without CSF-specific OCB at baseline, a 45-year-old SMA type 3 male patient (S04) and a 39-year-old SMA type 3 female patient (S09), positive OCB were found at a single time point during further treatment (d14 and m10, respectively) but no longer in subsequent CSF samples.

In general, the leukocyte count in the CSF ranged from 1 to 5/µL in 24 of 26 patients. Despite mainly being within the normal range, an increase in the leukocyte count was observed between d0 and the following time points, with the exception of m18, reaching statistical significance between d0 and d14 (*p* = 0.004), d0 and d28 (*p* = 0.024), d0 and d63 (*p* = 0.015), and d0 and m14 (*p* = 0.038). Summarizing OCB results, in 2 of 24 tested SMA patients, positive OCB were found at baseline. Throughout therapy, four SMA patients tested positive for OCB.

#### 3.2.2. CSF Total Protein, QAlbumin, and CSF Lactate

At baseline, seven SMA patients (28%) showed total protein values in the CSF above the upper reference level of 500 mg/L (range 502–588 mg/L), and six (25%) were found with a disturbed blood–CSF barrier function, five of those with an elevation of total protein. The patient with the highest total CSF protein levels in our study was a 57-year-old SMA type 3 male patient (S16). Total protein levels in this patient ranged from 583 mg/L (d0) to 907 mg/L (m14), rising from baseline to 817 mg/L at d63 and decreasing slightly to 742 mg/L at m6 and 624 mg/L at m10. From baseline on, QAlbumin values of this patient indicated CSF–blood barrier dysfunction at all time points (d0: 8.74 × 10^−3^; d63: 11.92 × 10^−3^; m6: 13.67 × 10^−3^; m10: 11.23 × 10^−3^; m14: 16.26 × 10^−3^). Other CSF parameters (cell count, lactate, OCB) were within the normal range. The remaining 18 patients had CSF protein levels within the reference range (211–477 mg/L). While there was no significant difference in total protein after the first injection of nusinersen, from d28 onward, significant changes were found (d0 to d28: 353 mg/L vs. 371 mg/L, *p* = 0.033; d0 to d63: 353 mg/L vs. 414.5 mg/L, *p* = 0.004; d0 to m6: 353 mg/L vs. 447 mg/L, *p* = 0.003; d0 to m10: 353 mg/L vs. 409 mg/L, *p* = 0.044). After m14, the increase persisted but was no longer significant. Fifteen patients (53.6%) were at least once above the upper reference level of 500 mg/L during therapy. After the first and second administration of nusinersen, no significant changes in QAlbumin were detected. We found significant changes in QAlbumin between d0 and d63 (4.85 × 10^−3^ vs. 5.77 × 10^−3^, *p* = 0.001), between d0 and m6 (4.85 × 10^−3^ vs. 5.79 × 10^−3^, *p* = 0.001), and between d0 and m10 (4.85 × 10^−3^ vs. 5.01 × 10^−3^, *p* = 0.041). Similar to changes in total protein, no further significant changes in QAlbumin were found from m14 onward. According to age-dependent adjustments for QAlbumin, 12 patients (42.9%) displayed CSF–blood barrier dysfunction at least once throughout therapy. Because of the very high CSF total protein values and corresponding QAlbumin values, we repeated the statistical analysis after excluding the above-mentioned SMA type 3 patient (S16). We then still found a significant difference in total protein values between d0 and d28 (Table S1, *p* = 0.033). QAlbumin values first differed significantly between d0 and d63 (Table S1, *p* = 0.001). A significant difference in the total protein and QAlbumin values was found until m6 (Table S1, total protein: *p* = 0.005; QAlbumin: *p* = 0.001). After excluding all SMA patients with a baseline dysfunctional CSF–blood barrier according to age-adjusted QAlbumin values (*n* = 5, here S00, S04, S06, S10, S19), we still detected a significant difference in both total protein (Table S2, *p* = 0.008) and QAlbumin (Table S2, *p* = 0.026) between d0 and d28. This significant difference remained present until m10 (Table S2; total protein: *p* = 0.01; QAlbumin: *p* = 0.025) and became insignificant thereafter. All 22 available lactate values at baseline were within the normal range between 1.26 and 1.96 mmol/L. During the treatment course, lactate levels were mainly stable but showed significant differences between d0 and m6 (*p* = 0.024), d0 and m10 (*p* = 0.034), and d0 and m18 (*p* = 0.028). Table 3 depicts the proportion of the CSF–blood barrier dysfunction according to age-adjusted reference levels for QAlbumin throughout nusinersen therapy.

### 3.3. Comparison of CSF Routine Parameters under Conventional and CT-Guided Lumbar Puncture

In 13 patients, CT-guided lumbar puncture was necessary due to scoliosis and previous spinal surgery. Lactate, CSF total protein, and QAlbumin values did not differ significantly in patients undergoing CT-guided LP compared to those undergoing conventional LP at baseline. At all following injections, there was no significant difference between the CSF leukocyte count, CSF total protein levels, and QAlbumin values in patients who underwent CT-guided lumbar puncture (interlaminar or transforaminal). Only lactate levels significantly differed between patients with conventional versus CT-guided LP at m10 (1.52 mmol/L vs. 1.28 mmol/L, *p* = 0.016) and m14 (1.63 mmol/L vs. 1.3 mmol/L, *p* = 0.009). Lactate levels ranged below the upper level of normal in both groups at both time points (conventional LP: m10: 1.22–1.88 mmol/L and m14: 1.37–2.06 mmol/L; CT-guided LP: m10: 1.17–1.43 mmol/L and m14: 1.18–1.41 mmol/L).

### 3.4. Association of CSF Protein Values and Clinical Parameters

No significant correlations between QAlbumin values at baseline (d0) and HFMSE, RULM, and 6MWT results were found. CSF total protein levels at baseline (d0) did not correlate with the HFMSE, RULM, and 6MWT. There was a positive correlation between QAlbumin at baseline (d0) and age (rho = 0.522, *p* = 0.009) as well as between total protein levels and age (rho = 0.548, *p* = 0.005). During the observation period (d0 to m18), there was no significant correlation between QAlbumin or total protein levels and the HFMSE, RULM, and 6MWT but a correlation of age with QAlbumin (rho = 0.326, *p* < 0.001) and total protein levels (rho = 0.326, *p* < 0.001). We found a significant association between QAlbumin values and total protein levels and SMA type (QAlbumin: rPB = 0.307, *p* < 0.001; total protein: rPB = 0.38, *p* < 0.001), sex (QAlbumin: rPB = 0.258, *p* = 0.003; total protein: rPB = 0.275, *p* = 0.001), *SMN2* copy number (QAlbumin: rPB = 0.183, *p* = 0.037; total protein: rPB = 0.269, *p* = 0.002), and modality of LP (QAlbumin: rPB = −0.274, *p* = 0.001; total protein: rho = −0.34, *p* < 0.001). Significantly higher total protein levels and higher QAlbumin values were found in SMA type 3 patients, in male patients, in patients with *SMN2* copy number ≥3, and in patients who underwent conventional lumbar puncture. QAlbumin values and total protein levels significantly correlated with procedure-related side effects (QAlbumin: rPB = −0.181, *p* = 0.046; total protein: rPB = −0.211, *p* = 0.019). Patients with lower CSF total protein levels or QAlbumin values experienced more procedure-related side effects. Overall, procedure-related side effects did not correlate with age, OCB status, and LP technique. However, a weak but significant association between sex and side effects was found: women reported procedure-related side effects in 50% of conducted LPs compared to 25.3% in men (chi-square = 9.321, phi = −0.254, *p* = 0.002). The linear mixed model analysis confirmed our former approach of correlating QAlbumin values with the LP method (Table 4; *p* = 0.022, F = 5.461) and age (Table 4; *p* = 0.009, F = 7.214), where we observed an additive effect (Table 4; *p* = 0.008, F = 7.308). All post hoc evaluations were abandoned due to the lack of sufficient data density to perform the estimated marginal means.

## 4. Discussion

The aims of this study were (1) to confirm recently published changes in CSF routine parameters in a representative cohort of adult nusinersen-treated SMA patients, (2) to assess a potential procedure-based effect caused by repeated LPs on CSF routine parameter changes under nusinersen treatment, and (3) to determine whether nusinersen is well tolerated according to CSF routine parameters in SMA patients with borderline-pathogenic CSF metrics at baseline. Overviewing a treatment period of a maximum of 18 months, we observed a significant elevation in CSF total protein levels and respective QAlbumin values in 38.9% of cases during the first 10 months of treatment, mainly without significant changes in lactate levels, leukocyte count, and OCB status, which is consistent with previously published data [11]. Elevated total protein levels and QAlbumin values indicate dysfunction of the CSF–blood barrier, which is found in numerous neurological diseases [21,22,23,24,25] or even without any apparent neurological disease [26]. In SMA patients, however, data on CSF total protein and QAlbumin values are sparse. In the past, CSF analysis was rarely performed in SMA patients since the diagnosis is confirmed by genetic testing. Wurster and colleagues reported elevated CSF total protein levels in 20% of their SMA cohort [11]. In our cohort, 28% of SMA patients had elevated total protein levels above the general reference interval of 500 mg/L at baseline. According to a recent study on age-related reference levels of total CSF protein, suggesting an upper reference level of 500 mg/L for 18–30 years of age and 600 mg/L for those at age 30 and above, most values seen in our cohort would still be within the normal range [27]. Only four patients (14.3%) had CSF protein levels above the age-adjusted upper reference level of 600 mg/L during treatment. While most SMA patients in our cohort had normal total CSF protein levels based on age-adjusted reference intervals, age-adjusted QAlbumin values indicated CSF–blood barrier dysfunction in 25% of SMA patients at baseline. In amyotrophic lateral sclerosis (ALS), another motor neuron disease, an elevation of CSF QAlbumin values was found in up to 20% of patients, which was seen as non-specific [28]. Thus, the origin of elevated QAlbumin values in treatment-naive SMA patients remains to be further elucidated and might be related to the neurodegenerative process. A comparative study between different motor neuron diseases, such as ALS or SMA, and healthy controls should be performed to further evaluate this phenomenon. A significant increase in the CSF protein levels and QAlbumin values during regular intrathecally administrated nusinersen might result either from nusinersen itself or its administration via repeated LP.

No clinical parameters influencing CSF protein parameters in SMA patients during the treatment course were identified in a previous study [11]. Consistently, we did not detect any correlation between motor function outcome measures (HFMSE, RULM, 6MWT) and CSF protein parameters both at baseline and during further treatment. However, significant associations between CSF total protein levels and/or QAlbumin values and SMA type, *SMN2* copy number, sex, and LP method were identified. An explanation is that CSF total protein levels and QAlbumin levels are age dependent and increase over time [27,29]. SMA type 3 and 4 patients in our cohort were older than SMA type 2 patients (median 33.5 vs. 43 years). Since SMA type 3 patients have typically more *SMN2* copies [4], patients with a higher *SMN2* copy number consequently displayed higher total protein levels and QAlbumin values. Patients who underwent conventional LP (higher number of SMA 3 patients) were older than patients who underwent CT-guided LP (mainly SMA type 2 patients; 34 vs. 46 years). Therefore, we suppose that the patients’ age mainly explains different findings for total protein levels and QAlbumin values, as previously described by Wurster et al. [11]. Multivariate analysis revealed that both age and the LP method influenced total protein levels and QAlbumin values in an additive manner. Therefore, in addition to age, which is well known to have an impact on CSF protein levels, the LP method also contributes to increases in total CSF protein levels and QAlbumin values. Higher total protein levels and QAlbumin values in men compared to women have been previously described [29], which is consistent with our findings. It is not known how far CSF changes during repeated LPs are influenced by gender. Our multivariate analysis showed no significant influence of gender on the course of total protein levels and QAlbumin values. QAlbumin values in our study were age-, but not sex adjusted. Castellazzi and colleagues found significant differences in QAlbumin values by a margin of 2.2 units for men [30]. Since revised sex-adjusted reference intervals for total protein levels and QAlbumin values have not been established, we are not able to completely rule out a sex-specific effect on changes in CSF protein parameters after repeated LPs. The interval between each LP might be a relevant factor since significant changes in protein elevation were detected only at the beginning of nusinersen treatment (until month 10), when therapy intervals were shorter compared to the maintenance period (14 to 35 days vs. 4 months). This finding remains after adjusting for SMA patients with CSF–blood barrier dysfunction at baseline, which is consistent with the previously reported elevation of CSF total protein levels found in nusinersen-treated SMA patients with normal CSF routine parameters at baseline [31]. Therefore, we suggest that a short interval between repeated LPs is more likely to result in an elevation of CSF protein parameters, especially against the background of numerous repeated LPs. An adult SMA patient cohort undergoing repeated LP at the same time points and without administration of any drug would have served best to address these questions, but this approach would not be ethically justified any longer. CSF lactate values differed significantly between d0 and m6, m10, and m18 but stayed stable within the normal range throughout nusinersen therapy. Lactate values in the CSF serve to differentiate bacterial from viral meningitis [32], but no patient in our cohort demonstrated either further suspect CSF findings or clinical features of meningitis. Therefore, we consider these changes as non-specific.

Our results contain important information for the new field of gene-specific therapies. Ongoing studies on ASO treatment for genetic variants of ALS [33,34,35] will further elucidate how repeated LPs potentially change CSF protein parameters.

At baseline, in 28.6% of SMA patients, at least one abnormal CSF parameter was detected (7.1% with positive OCB, 25% with CSF–blood barrier dysfunction). In a previously published study, positive OCB were found in 3.9% and CSF–blood barrier disorder in 24.6% of nusinersen-treated SMA patients at baseline [11]. In our study, SMA patients with CSF–blood barrier dysfunction at baseline did not report side effects more frequently than SMA patients with normal QAlbumin values. The OCB status was not related to side effects, either. However, two SMA patients of our cohort developed CSF-specific OCB under treatment with nusinersen, though this finding was temporary. No data are available regarding incidental findings of positive OCB in SMA. Considering other motor neuron diseases, positive OCB were detected in 3.5% of patients with ALS [36]. Therefore, positive OCB rather seem to be incidentally present in different motor neuron diseases such as SMA, since both patients with positive OCB findings at baseline in our study displayed no signs of infectious or autoimmune CNS disease. The transient appearance of CSF-specific OCB observed in two SMA patients in our study may be attributable to nusinersen treatment, though further investigation is necessary to determine a possible CSF immune reaction against nusinersen. Altogether, SMA patients with abnormal CSF routine parameters did not report side effects more frequently. None of our patients reported any symptoms of increased intracranial pressure indicating hydrocephalus. However, we did not perform further cranial imaging to rule out asymptomatic hydrocephalus. Further diagnostics of hydrocephalus, i.e., cranial imaging or measuring CSF opening pressure, could give further information regarding the possible development of asymptomatic hydrocephalus in patients undergoing repeated LP, especially in nusinersen-treated SMA patients [37].

## 5. Conclusions

This study confirms the previous findings on mild changes in CSF routine parameters in adult SMA patients under nusinersen treatment. Our results confirm the notion that intrathecal administration of nusinersen in adult SMA patients is generally safe and well tolerated. Conspicuous pre-treatment CSF findings such as OCB had no impact on the tolerability of nusinersen. Considering ongoing studies assessing intrathecal administration of ASOs in other neurological diseases, it is important to further investigate and monitor CSF routine parameters.

## Figures and Tables

**Table 1:** Demographic characteristics of SMA patient groups. Age, disease duration until therapy start, HFMSE, 6MWT, and RULM are described as median with range. Above-listed values for the HFMSE, 6MWT, and RULM were collected at baseline (d0). The *SMN2* copy number was not available in one 58-year-old SMA type 3 male patient. 6MWT: 6-minute walk test; CNS: central nervous system; CT: computed tomography; HFMSE: Hammersmith Functional Motor Scale Expanded; LP: lumbar puncture; OCB: oligoclonal bands; RULM: Revised Upper Limb Module; SMA: spinal muscular atrophy; *SMN*2 gene: survival motor neuron 2 gene.

	SMA (Total)	SMA 2	SMA 3/4
*n*	28	10	18
Women	10 (35.7%)	4 (40%)	6 (33.3%)
Age, years (range)	36 (19–65)	34 (20–51)	39 (19–65)
SMN2 copy ≥3	15 (53.6%)	1 (10%)	14 (82.4%)
Disease duration, years (range)	32.5 (2–61.5)	33.25 (19.42–50)	31.75 (2–61.5)
HFMSE	9.5 (0–63)	0.5 (0–11)	35 (3–63)
6MWT (m)	390 (0–512)	0	390 (42–512)
RULM	18.5 (0–37)	9.5 (0–21)	29 (12–37)
CT-guided LP	13 (46.4%)	10 (100%)	3 (16.7%)
Abnormal CSF leukocyte count at baseline (%)	0 (0%)	0 (0%)	0 (0%)
Abnormal CSF lactate at baseline (%)	0 (0%)	0 (0%)	0 (0%)
Abnormal CSF–blood barrier dysfunction at baseline (%)	6 (21.4%)	2 (20%)	4 (22.2%)
Positive OCB at baseline (%)	2 (7.1%)	2 (20%)	0 (0%)

**Table 2:** Overview of CSF routine parameters at the nusinersen application time points (d0 to m22). Values for time point m22 were not further analyzed. Values are presented as the median and range. *n* describes the number of analyzed samples. CSF: cerebrospinal fluid; d: day; m: month; QAlbumin: CSF/serum albumin quotient.

Time Point	d0	d14	d28	d63	m6	m10	m14	m18	m22
CSF leukocyte count (cells/µL)	1.0 (0.0–4.0)	1.0 (0.0–33.0)	1.0 (0.0–12.0)	2.0 (0.0–5.0)	1.0 (0.0–3.0)	1.0 (0.0–3.0)	1.0 (0.3–4.0)	0.0 (0.0–2.0)	1.0 (1.0–1.0)
	*n* = 26	*n* = 25	*n* = 23	*n* = 25	*n* = 23	*n* = 18	*n* = 13	*n* = 7	*n* = 2
CSF lactate (mmol/L)	1.5 (1.26–1.96)	1.45 (1.17–1.96)	1.42 (1.25–1.72)	1.44 (1.27–1.79)	1.38 (1.24–1.92)	1.42 (1.17–1.88)	1.41 (1.18–2.06)	1.28 (1.16–1.61)	1.32 (1.20–1.43)
	*n* = 22	*n* = 18	*n* = 16	*n* = 20	*n* = 19	*n* = 16	*n* = 11	*n* = 7	*n* = 2
CSF total protein (mg/L)	353 (211–588)	367 (211–587)	371 (210–583)	414.5 (198–817)	447 (258–748)	409 (187–624)	375 (173–907)	415.5 (239–606)	266 (214–318)
	*n* = 25	*n* = 19	*n* = 15	*n* = 22	*n* = 18	*n* = 17	*n* = 13	*n* = 6	*n* = 2
QAlbumin (×10^−3^)	4.85 (1.6–9.04)	4.82 (2.73–10.57)	4.79 (1.94–9.46)	5.77 (2.88–11.92)	5.79 (3.4–13.67)	5.01 (1.83–11.23)	4.41 (1.76–16.26)	5.33 (1.98–8.94)	2.54 (1.88–3.2)
	*n* = 24	*n* = 19	*n* = 14	*n* = 23	*n* = 18	*n* = 17	*n* = 13	*n* = 6	*n* = 2

**Table 3:** SMA patients with or without CSF–blood barrier dysfunction according to age-adjusted reference levels for QAlbumin throughout therapy (d0 to m22). CSF: cerebrospinal fluid; d: day; m: month; SMA: spinal muscular atrophy.

Time Point	*n*	CSF–Blood Barrier Dysfunction	
		Yes	No
d0	24	6 (25%)	18
d14	19	3 (15.8%)	16
d28	14	1 (7.1%)	13
d63	22	7 (31.8%)	15
m6	18	7 (38.9%)	11
m10	17	5 (29.4%)	12
m14	13	3 (23.1%)	10
m18	6	2 (33.3%)	4
m22	2	0	2

**Table 4:** Predictors of CSF total protein and QAlbumin values. A mixed linear model was used. *Time point* refers to nusinersen injection (d0, d14, d28, d63, m6, m10, m14, m18). *LP method* refers to CT-guided or conventional LP. CSF: cerebrospinal fluid; LP: lumbar puncture; QAlbumin: CSF/serum albumin quotient.

	CSF Total Protein as a Dependent Variable				QAlbumin as a Dependent Variable			
	Numerator df	Denominator df	F	*p* Value	Numerator df	Denominator df	F	*p* Value
Sex	1	82	0.444	0.507	1	83	0.456	0.501
Age	1	82	0.444	0.834	1	83	7.214	0.009
Time point	7	82	1.83	0.092	7	83	1.421	0.208
LP method	1	82	0.569	0.453	1	83	5.461	0.022
Time point and sex	7	82	0.256	0.969	7	83	0.332	0.937
Time point and age	7	82	1.634	0.137	7	83	1.367	0.23
Time point and LP method	7	82	0.228	0.977	7	83	0.566	0.782
LP method and age	1	82	0.00	0.995	1	83	7.308	0.008
Time point and sex and age	7	82	0.105	0.998	7	83	0.334	0.936
Time point and sex and LP method	7	82	1.093	0.375	7	83	0.974	0.456
Time Point and age and LP method	6	82	0.25	0.958	6	83	0.747	0.613

## Data Availability

All data generated or analyzed during this study are included in this published article.

## Supplementary Materials

The following are available online at https://www.mdpi.com/2076-3425/11/3/296/s1: Table S1: Overview of CSF routine parameters during therapy (d0 to m22); Table S2: Overview of CSF routine parameters during therapy (d0 to m22).
