# Supplementary material for: Cerebrospinal Fluid Parameters in Antisense Oligonucleotide-Treated Adult 5q-Spinal Muscular Atrophy Patients
Source: Brain Sci. 2021 Feb 26;11(3):296. doi: 10.3390/brainsci11030296 (PMC7996901; doi:10.3390/brainsci11030296)
Supplement: Supplementary file 1 [file brainsci-11-00296-s001.pdf]

Time point		d0	d14	d28	d63	m6	m10	m14	m18	m22
CSF total protein (mg/L)		344 (211-588)	367 (211-587)	371 (210-583)	404 (198-704)	433 (258-748)	403.5 (187-572)	349 (173-660)	415.5 (239-606)	266 (214-318)
		n=24	n=19	n=15	n=21	n=17	n=16	n=12	n=6	n=2
QAlbumin ($x 10^{-3}$)		4.79 (1.6-9.04)	4.82 (2.73-10.57)	4.785 (1.94-9.46)	5.63 (2.88-9.84)	5.71 (3.4-9.98)	4.995 (1.83-8.11)	4.205 (1.76-10.66)	5.33 (1.98-8.94)	2.54 (1.88-3.2)
		n=23	n=19	n=14	n=22	n=17	n=16	n=12	n=6	n=2

Table S1. Overview of CSF routine parameters during therapy (d0 to m22). The SMA patient with the highest CSF total protein levels throughout therapy (S16) was excluded. Values for time point m22 were not further analyzed. Values are presented as median and range. N describes number of analyzed samples. CSF: cerebrospinal fluid. d: day. m: month. QAlbumin: CSF/serum quotient of Albumin.

Time point	d0	d14	d28	d63	m6	m10	m14	m18	m22
CSF total protein (mg/L)	329 (211-587)	353 (211-587)	379 (210-583)	388 (198-704)	399 (258-748)	389 (187-522)	306 (173-545)	338.5 (239-436)	266 (214-318)
	n=19	n=16	n=14	n=18	n=14	n=13	n=9	n=4	n=2
QAlbumin ($x 10^{-3}$)	4.025 (1.6-7.98)	4.32 (2.73-7.28)	4.63 (1.94-9.46)	5.43 (2.88-8.94)	5.595 (3.4-9.98)	4.92 (1.83-7.11)	3.95 (1.76-5.43)	4.03 (1.98-5.83)	2.54 (1.88-3.2)
	n=18	n=16	n=13	n=19	n=14	n=13	n=9	n=4	n=2

Table S2. Overview of CSF routine parameters during therapy (d0 to m22). Patients with elevated CSF total protein and/or CSF-blood-barrier dysfunction according to age-adjusted QAlbumin values are excluded (S0, S4, S6, S10, S19) Values for time point m22 were not further analyzed. Values are presented as median and range. N describes number of analyzed samples. CSF: cerebrospinal fluid. d: day. m: month. QAlbumin: CSF/serum quotient of Albumin.
